# Case report: Giant coronary artery aneurysms with severe stenosis and multiple abdominal artery aneurysms

**DOI:** 10.3389/fmed.2023.1187690

**Published:** 2023-05-25

**Authors:** Hongli Gao, Hongwei Li

**Affiliations:** Department of Cardiology, Cardiovascular Center, Beijing Friendship Hospital, Capital Medical University, Beijing, China

**Keywords:** giant coronary artery aneurysms, abdominal artery aneurysms, stenosis, Kawasaki disease, coronary artery bypass grafting

## Abstract

**Background:**

Giant coronary artery aneurysms (GCAAs) were relatively rare. Little was known about its characteristics, its etiology and its therapy. GCAAs with multiple abdominal artery aneurysms (AAAs) were more unusual and rarer.

**Case presentation:**

A 29-year-old female presented to our hospital with abrupt-onset abdominal pain in the left upper quadrant and then she died in 2018. In 2016, prior to this visit, she came to our department for intermittent retrosternal compression pain during rest or sports activities. Medical history showed she had a coronary artery aneurysm (CAA) in 2004. We found evidence of multiple coronary aneurysms with severe stenosis and multiple AAAs and coronary artery bypass grafting (CABG) was carried out. In combination with laboratory analysis, imaging studies, and pathological examination, CAA may result from the long-term effects of Kawasaki disease (KD). Finally, the patient died of a ruptured abdominal aneurysm.

**Conclusions:**

We report a rare case of GCAAs with severe stenosis and multiple AAAs in a young woman with a history of KD-induced coronary aneurysm. Although the understanding of the optimal treatment strategy for GCAAs combined with multiple aneurysms was limited, we found that CABG was effective in the treatment of GCAAs in this patient. In the clinical treatment of patients with GCAAs, attention should be paid to the examination of systemic blood vessels.

## Background

Coronary artery aneurysm (CAA) is defined as a coronary artery dilatation exceeding the diameter of the normal adjacent segment or the diameter of the patient's largest coronary vessel diameter by 1.5 time (1), while a standard diameter of more than 20 mm is widely used to define a giant coronary artery aneurysm (GCAA) (2). It was rare in clinical findings, with the overall incidence of coronary artery aneurysm ranging from 0.3 to 5.3% (3), and the prevalence of GCAA was reported as 0.02%. Patients with GCAA who also have multiple abdominal diseases are extremely rare, and experience with the associated treatment is even less. Some case reports have found that CABG may be effective (4). Herein, this study present the case of a 29-year-old Chinese female with coronary aneurysms with severe stenosis and multiple abdominal aneurysms and discuss related treatment options.

## Case presentation

A 29-year-old female presented to our department in October 2016. The patient experienced intermittent retrosternal pressing pain at rest or during physical activities that lasted a few minutes to half an hour. Medical history showed this patient had a fever at 5 years old (missing medical records). In 2004, the patient presented to a local Secondary hospital with retrosternal chest pain after physical activities. Coronary angiography (CAG) was performed, and the result showed two aneurysmal dilatations in the proximal segment of the left anterior descending (LAD) artery, one aneurysmal dilatation in the mid-segment of the right coronary artery (RCA), and 100% occlusion at the end of left circumflex artery posterior descending branch (LCXp; due to the length of time and the limitations of the local hospital, imaging data was missing at the time). The patient had no prior risk factors for atherosclerosis. The patient was diagnosed with CAA and was treated with oral aspirin (0.3 g tid for the first 5 days, 0.1 g tid for the following 3 weeks and 0.1 g qd thereafter) for 6 months. According to the patient and her family, the patient's symptoms are in remission. Due to the limitation of the local hospital's diagnosis and treatment capacity, no follow-up and treatment were performed.

**Figure 1:**
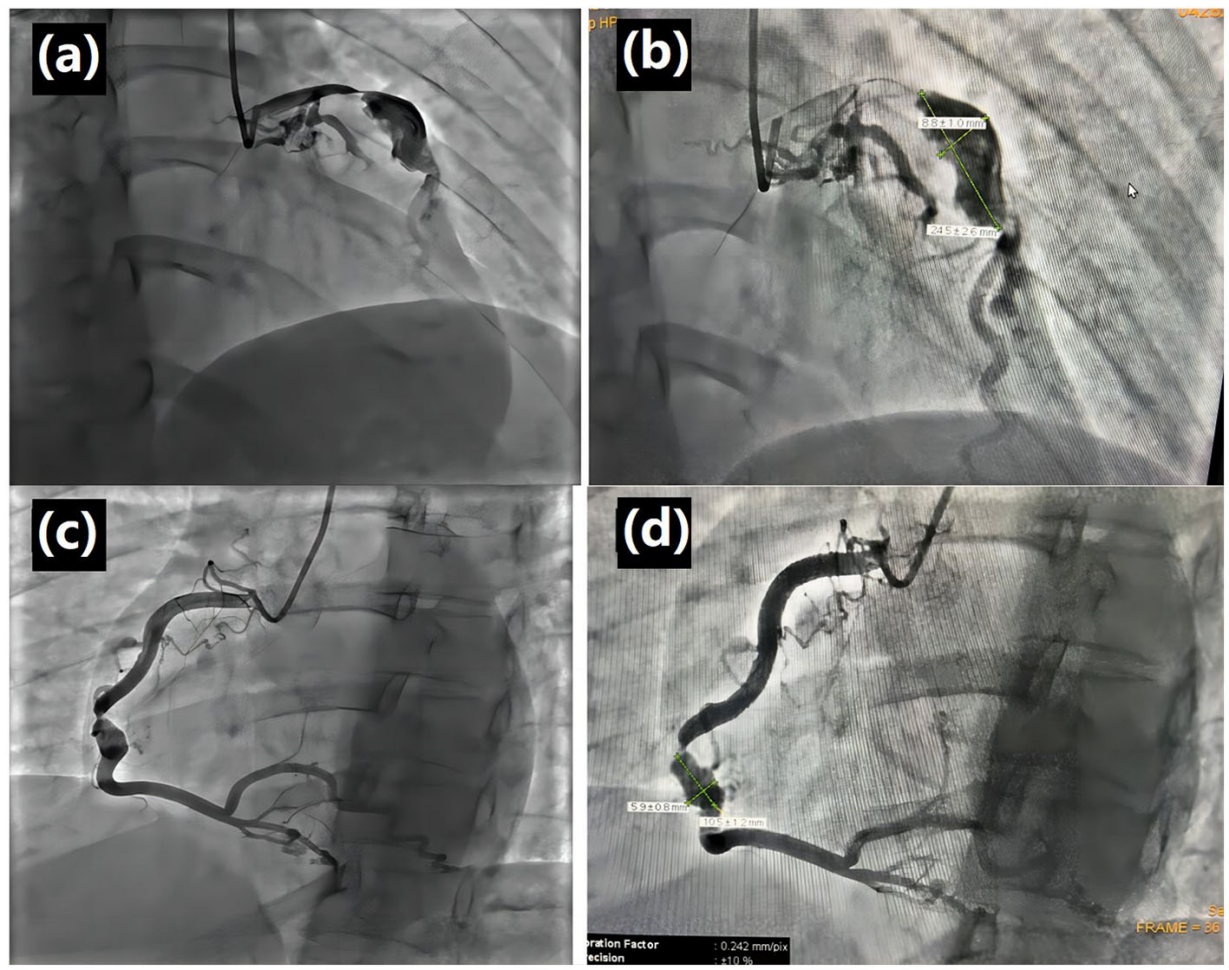
Left coronary angiography in the caudal view and right coronary angiography in the left-anterior oblique and cranial view. **(a)** Left coronary angiography in the caudal view showed a severe stenosis of the proximal segment of left anterior descending (LAD), following by a huge coronary aneurysm at the proximal middle segment of LAD. **(b)** Transversal diameter of 8.8 ± 1.0 mm and longitudinal diameter of 24.5 ± 2.6 mm. **(c)** Right coronary angiography in the left-anterior oblique and cranial view showed a severe stenosis of the middle segment of right coronary artery (RCA), following by a huge coronary aneurysm at the middle segment of RCA. **(d)** Transversal diameter of 5.9 ± 0.8 mm and longitudinal diameter of 10.5 ± 1.2 mm.

**Figure 2:**
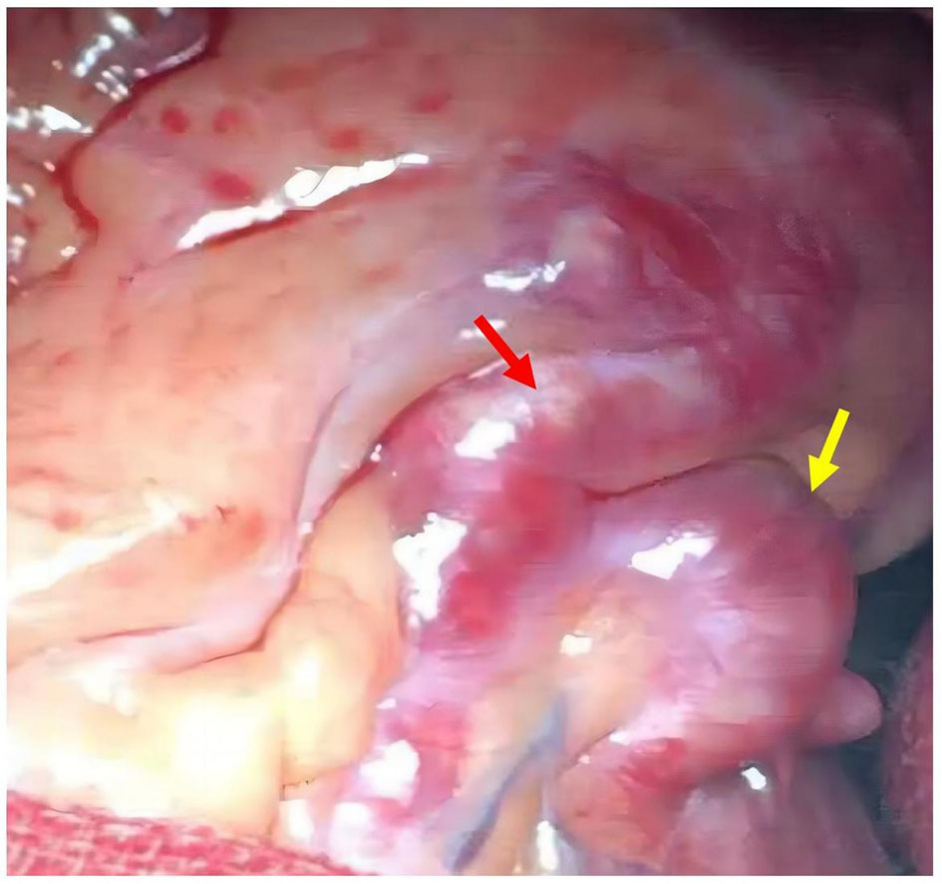
Huge coronary aneurysm observed during coronary artery bypass grafting (CABG) surgery; left anterior descending (LAD) aneurysm (red arrow); D1 aneurysm (yellow arrow).

**Figure 3:**
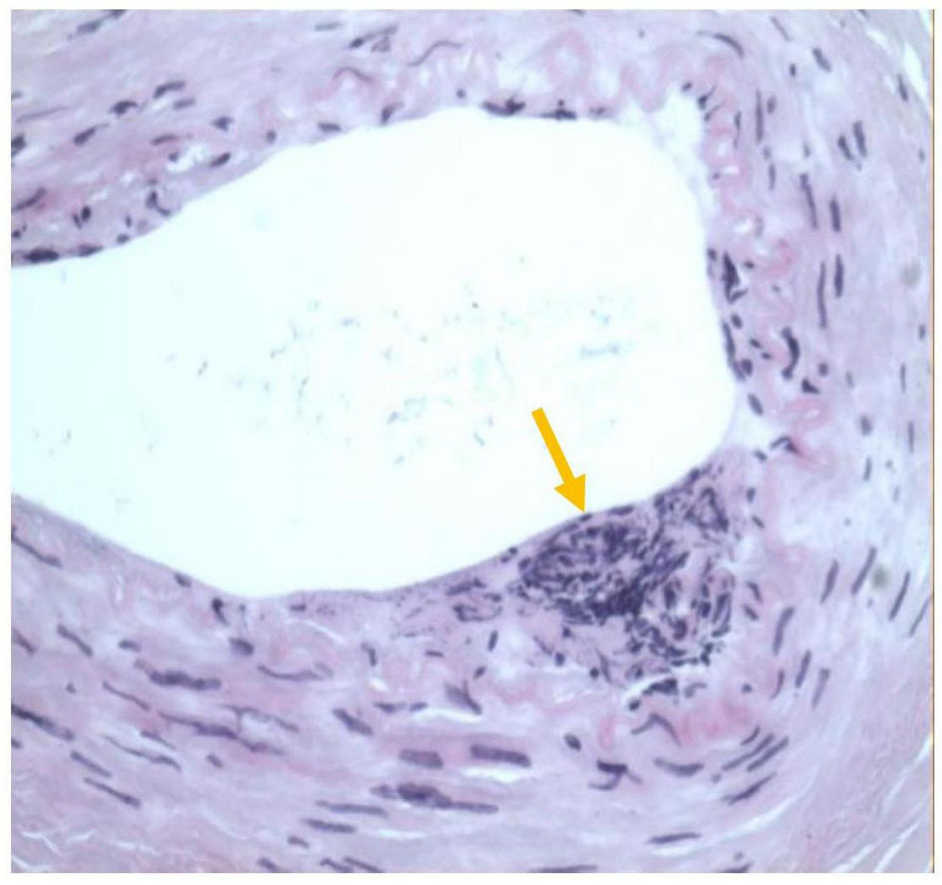
Pathological findings of left internal mammary artery (LIMA).

During this visit, the patient reported intermittent retrosternal compression pain at rest or during exercise lasting for several minutes to half an hour. CAG was performed, and the results showed a sizeable aneurysmal dilatation in the LAD with 95% localized stenosis, 100% occlusion of the LCXp, and a sizeable aneurysmal dilatation of the RCA with 90% localized stenosis (Figure 1). Abdominal ultrasound was also performed, and the result showed localized expansion of the hepatic artery with a maximum width of ~1.5 cm, which was considered as a hepatic arterial aneurysm. Peripheral blood vessel ultrasound showed no abnormality. The lab test results found that patient's blood glucose levels and blood lipid levels were in the normal ranges. All levels of indicators of autoimmune-related diseases, such as erythrocyte sedimentation rate, C-reactive protein, immunoglobulin, complement, antinuclear antibody, were in the normal ranges. The patient was diagnosed with CAAs with severe stenosis and multiple AAAs. Because a ruptured aneurysm can cause life-threatening bleeding, coronary artery bypass grafting (CABG) was carried out. Two giant coronary aneurysms were seen during surgery (Figure 2). The pathological examination of the left internal mammary artery (LIMA) confirmed minor lesions of endovascular inflammation in the LIMA and fibroproliferative degeneration around the vessel wall (Figure 3). Combined with the patient's previous medical history, the aneurysm may be induced by Kawasaki disease (KD). Oral aspirin (0.1g qd) and atorvastatin (20 mg qn) were prescribed, and the patient did not report any chest pain after the treatment. Four months after the CABG, coronary computed tomography angiography (CTA) showed that CABG was done and LIMA-LAD graft patented, local aneurysmal dilatation of the RCAm with eccentric stenosis, LADp-m with 60% localized stenosis and partial aneurysmal dilatation. The lumens of LCX and OM were small.

**Figure 4:**
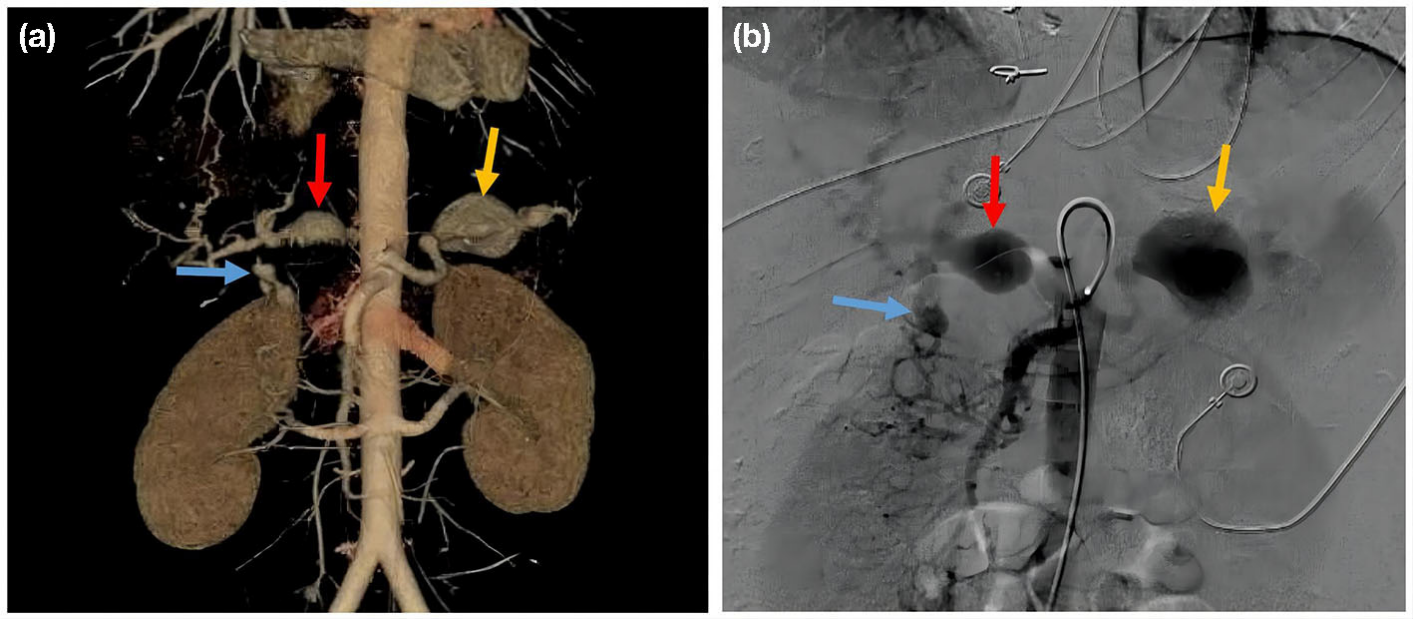
**(a)** Aortic CT angiography; splenic artery aneurysm (yellow arrow); common hepatic artery aneurysm (red arrow); gastroduodenal artery aneurysm (blue arrow). **(b)** Celiac artery angiography, splenic artery aneurysm (yellow arrow); common hepatic artery aneurysm (red arrow); gastroduodenal artery aneurysm (blue arrow).

After discharge, the patient followed the doctor's advice and insisted on treatment. However, in March 2018, the patient presented with abrupt-onset abdominal pain in the left upper quadrant. CTA revealed multiple aneurysms in the abdominal cavity involving the splenic artery, common hepatic artery, hepatic artery, gastroduodenal artery, and superior pancreaticoduodenal artery. The largest one was 3.6 cm × 3.7 cm × 3.7 cm, and it was located in the splenic artery (Figure 4a). Abdominal angiography showed aneurysmal-like structures in the hepatic artery, splenic artery, and gastroduodenal artery (Figure 4b), but no spillover of contrast medium was noted. We wanted to perform an interventional therapy, but the patient's family refused. Then the patient died suddenly after 3 days. The abdominal ultrasound examination showed effusion in the abdominal cavity, which was considered to be caused by a ruptured aneurysm.

## Discussion

The predominant cause of CAA is atherosclerosis. Other less frequent etiologic factors are congenital abnormalities, Kawasaki disease, trauma, Ehlers-Danlos syndrome, Marfan syndrome, poliarteritis nodosa, Takayasu disease, and syphilis (5). Despite that previous studies found that atherosclerosis accounts for >90% of coronary artery aneurysms in adults (6). However, this patient was young and had no risk factors for atherosclerosis. This patient had a history of coronary artery disease for more than 10 years. Ultrasonography at admission did not reveal atherosclerotic manifestations in the peripheral blood vessels. Therefore, aneurysmal lesions caused by atherosclerosis were excluded. LIMA pathology after CABG showed endocarditis. Hence, a congenital CAA was excluded. Examination during hospitalization did not support the diagnosis of connective tissue disease. Considering that vasculitis without atherosclerosis can cause coronary aneurysms in patients with Kawasaki disease (7), the diagnosis of KD-based CAA was made a posteriori on the basis of these findings.

GCAAs with multiple AAAs are rare. There have only been a few reports of cases with GCAAs and multiple AAAs. Su et al. documented a female infant presented to the hospital at 2 months of age with a giant aneurysm of the right coronary artery, with multiple small aneurysms of the left coronary artery caused by KD (8). Jiang et al. (9) reported a 62-year-old female patient with right coronary aneurysm with concomitant abdominal aorta as well as right renal artery aneurysm caused by KD. Schukraft et al. recently reported a case of a 78-year-old Caucasian male with a 5 × 4 cm giant aneurysm of the right coronary artery and a rapidly expanding AAA, and no diagnosis was given in that article. Aorto-bi-iliac bypass grafting and CABG of the right coronary artery was performed, and the patient recovered well after the surgical procedure (4). Our case was very unusual. First of all, GCAAs with multiple AAAs were rarely seen in young female adults. In addition, the coronary artery aneurysms in this case were giant and were accompanied by multiple abdominal aneurysms, which were rarely reported. Moreover, though it is not a systematic part of the routine and guidelines, CTA was performed and revealed multiple aneurysms in the abdominal cavity, such as celiac artery aneurysm, splenic artery aneurysm, common hepatic artery aneurysm, gastroduodenal artery aneurysm, which eventually lead to patient death.

Based on the clinical data, this patient's GCAAs with AAAs were most likely due to KD. CAA occurred in up to 25% of KD patients due to delayed diagnosis and treatment (10). About 5% of KD patients may develop CAA even after early diagnosis and administration of intravenous immunoglobulin and oral aspirin (11). The safety and efficacy of regimens for KD-related coronary thrombosis have not been validated by randomized clinical trials so far. However, the 2013 Japanese Circulation Society guideline and the 2017 American Heart Association (AHA) guideline both recommended using antiplatelet drugs, such as aspirin, for the acute phase before pseudo-normalization of the aneurysm was achieved (12, 13). The 2017 AHA guidelines stated that dual antiplatelet therapy could also be considered in some situations. There has been no consensus on when antiplatelet drugs should be stopped, nor on the optimal dose or duration in adult patients with KD-related cardiovascular sequelae (13). According to the latest American College of Rheumatology/Vasculitis Foundation Guideline for the Management of Kawasaki Disease, the use of intravenous immunoglobulin (IVIG) with other adjuvant agents for patients with KD and CAA (14). Long-term aspirin therapy as secondary prevention for patients with a history of >4.0 mm aneurysms may reduce adverse outcomes. Anticoagulation therapy using warfarin or low-molecular-weight heparin is currently recommended for patients with a large CAA or thrombosis. Some KD patients may benefit from statins, but it is not clear which subtype of patients will benefit from statins (15). In this case, the patient had been taking aspirin for only half a year after being diagnosed with KD 12 years earlier. Due to the lack of understanding of CAAs with multiple AAAs in China, no regular follow-up assessments of the progression of the CAA were completed. Long-term antiplatelet, anticoagulant, and statin therapy, along with regular follow-up of CAA imaging, may improve patient outcomes.

Non-pharmacological interventions such as percutaneous coronary intervention (PCI), CABG, and cardiac transplantation were also commonly used to treat large aneurysms (15). There was no general consensus regarding the treatment of CAAs with multiple AAAs caused by KD. Some studies found a better short-term effect but a higher reintervention rate with PCI compared with CABG (16). Others found that CABG was superior to PCI in patients with multivessel lesions (17). Due to the unique anatomical structure and location of KD-associated aneurysms, restenosis, thrombosis, competing native blood flow, and occlusion may occur after CABG. The reintervention rate was higher after CABG, especially in patients without ischemic symptoms before the intervention, due to the native coronary artery competitive flow (18). Therefore, the choice of PCI or CABG depended mainly on the condition of the involved vessels. In this patient, PCI may have failed due to underestimation of lumen diameter and inappropriate stent size due to severe stenosis of the GCAAs in both coronary arteries. Hence, CABG was implemented instead of PCI. The patient reported no recurrence of chest pain after CABG, and CTA showed that the surgery was successful.

The optimal timing of intervention for AAAs is currently unclear. Studies have shown that it is reasonable to apply dynamic monitoring in asymptomatic patients with visceral aneurysms <2.0 cm. However, timely interventions such as endovascular embolization and bypass grafting were needed to prevent aneurysm rupture if the growth of the aneurysm accelerated and severe pain occurred during dynamic monitoring (19). This patient had GCAAs combined and multiple AAAs, which required dynamic monitoring and timely interventions. However, the patient's relatives rejected the treatment, and the patient died. Patients with GCAAs should be considered for the potential risk of AAAs, and peripheral blood vessels should be screened.

## Conclusions

We reported a rarely-seen case of GCAAs with multiple AAAs. Its characteristics and etiology are in favor of KD-induced coronary aneurysms, and there is no consensus regarding the optimal approach to the CAA. For patients with severe coronary artery involvement, systemic screening for abdominal aneurysms was necessary because of the strong association between these two diseases. The understanding and treatment of adult KD should be emphasized. Standardized treatment and regular follow-up were critical for patients with KD.

## Data availability statement

The original contributions presented in the study are included in the article/supplementary material, further inquiries can be directed to the corresponding author.

## Ethics statement

The studies involving human participants were reviewed and approved by the Institutional Review Committee of Beijing Friendship Hospital. The patients/participants provided their written informed consent to participate in this study. Written informed consent was obtained from the individual(s) for the publication of any potentially identifiable images or data included in this article.

## Author contributions

HG completed the writing of the paper. HL provided patient information. HG and HL made revisions to the paper. Both authors confirmed the final version of the paper.
